# Genomics has more to reveal

**DOI:** 10.18632/oncotarget.28596

**Published:** 2024-06-20

**Authors:** Laurène Fenwarth, Nicolas Duployez

**Keywords:** acute myeloid leukemia, genomics, UBTF

Molecular and cytogenetic analyses are now used to identify mutations and structural variants defining distinct subtypes of acute myeloid leukemias (AML) and myelodysplastic syndromes (MDS). These genetic considerations have become essential for risk stratification and the selection of appropriate treatments, including the use of allogeneic hematopoietic stem cell transplantation. Despite over 15 years of genomic research since the first publication of the AML genome [1] and large studies like The Cancer Genome Atlas (TCGA) [2], around 15% of AML cases remained genetically unclassifiable with current knowledge [3–5] Notably, several studies in both adults and children identified a subset of AML without known initiating events but particularly enriched in *FLT3*-ITD and *WT1* mutations, and normal karyotypes with an overall unfavorable prognosis [6, 7].

In 2021–2022, notably thanks to advancements in bioinformatic approaches and tools, recurrent somatic tandem duplications (TD) of a portion of the *UBTF* gene were identified in high-risk pediatric AML cases [8, 9]. With increased screenings of retrospective cohorts, the characteristics associated with this molecular alteration have since been confirmed. *UBTF*-TD are considered initiating events in leukemogenesis and define a distinct entity of myeloid malignancies.

It is estimated that *UBTF*-TD affect 4% of pediatric AML cases (9% of relapsed pediatric AML cases) [9], 3% of young adult AML cases (ages 18–60 y), and less than 0.5% of cases after age 60 y [10, 11]. These studies have also demonstrated a close continuum with MDS. In a recent series of adult AML, 20% of cases with *UBTF*-TD were secondary to an MDS [11]. The study of pediatric MDS cases without genetic predisposition found a frequency of about 30% for *UBTF*-TD mutations [12]. *UBTF*-TD is associated with a specific pattern of additional genetic lesions, including *WT1* mutations (∼50%), *FLT3*-ITD (∼50%), and trisomy 8 (∼30%, while ∼60% of cases have a normal karyotype), and is mutually exclusive with other class-defining lesions (i.e., *NPM1* or *CEBPA*-bZIP mutations and recurrent fusions).

The prognosis for MDS/AML with *UBTF*-TD is overall poor for a generally young patient population [9–11]. Most of the available data, coming from AML cases treated with intensive chemotherapy ± allogeneic hematopoietic stem cell transplantation showed higher rates of induction failures and relapses and, when evaluated, higher rates of minimal residual disease (MRD) following treatment compared to UBTF-wild-type patients [9].

The recognition of these cases, previously genetically unclassifiable, paves the way for their detection in routine practice and the development of new therapeutic strategies. In particular, transcriptional studies demonstrated a high expression of HOXA/HOXB cluster genes and MEIS1, a signature also found in *NPM1*-mutated AML and suggesting sensitivity to menin inhibitors. A recent study has confirmed this sensitivity in primary AML cells with *UBTF*-TD [13] Considering the strong association of this alteration with *FLT3*-ITD, combinations with FLT3 inhibitors also require further evaluation.

If there was any doubt, this discovery demonstrates that genomics, extensively deployed over the past two decades, still has much to reveal to us.
